# Comparison of Internal and Marginal Adaptation of Endocrowns at Different Depths Fabricated by the Digital and Conventional Impression Techniques: Internal and Marginal Fit of Endocrowns

**DOI:** 10.1155/2024/5526272

**Published:** 2024-11-07

**Authors:** Fatemeh Razavi Ardekani, Hamid Neshandar Asli, Naghme Musapoor, Mehran Falahchai

**Affiliations:** ^1^Department of Prosthodontics, School of Dentistry, Yasuj University of Medical Sciences, Yasuj, Iran; ^2^Department of Prosthodontics, Dental Sciences Research Center, School of Dentistry, Guilan University of Medical Sciences, Rasht, Iran; ^3^Department of Prosthodontics, Dental Sciences Research Center, School of Dentistry, Qazvin University of Medical Sciences, Qazvin, Iran

**Keywords:** computer-aided design, dental bonding, dental internal adaptation, dental marginal adaptation

## Abstract

**Introduction:** Sufficient information is not available regarding the comparative accuracy of digital and conventional impression techniques at different depths for the fabrication of endocrown restorations. Thus, this study aimed to compare the marginal (M) and internal (I) adaptation of endocrowns at different depths fabricated by the digital and conventional impression techniques.

**Materials and Methods:** In this in vitro study, two endodontically treated molar teeth with 2- and 4-mm cavity depths were used for the fabrication of endocrowns. Conventional and digital impressions were made from each tooth 17 times. A total of 68 zirconia endocrowns were fabricated and seated, and their I and M adaptation was assessed by the silicone replica technique. Data were statistically analyzed.

**Results:** The M and I gaps at 2- and 4-mm cavity depths were significantly smaller in the digital, than the conventional, impression technique (*p*  < 0.05). Increasing the cavity depth significantly increased the M and I gaps only in the digital technique (*p*  < 0.05). The largest gap in all groups was noted in the pulpal (P) region (*p*  < 0.05). The smallest gap was found in the M and cervical (C) regions in the conventional groups with 2- and 4-mm cavity depths, and the digital group with 4 mm cavity depth, and in the M region in the digital group with 2 mm cavity depth (*p*  < 0.05).

**Conclusion:** Unlike the conventional impression technique, the M and I adaptation decreased by an increase in cavity depth in the digital technique; nonetheless, the digital impression technique still showed higher M and I adaptation than the conventional technique.

## 1. Introduction

The long-term clinical success of severely damaged endodontically treated teeth depends on the type and quality of restoration for maximum preservation of the sound tooth structure and minimal microleakage [1]. Core reconstruction with a post extended into the canal along with a full-coverage coronal restoration is a well-accepted commonly practiced technique for endodontically treated teeth [2]. Nonetheless, post space preparation of the canal is associated with further removal of the tooth structure and increases the risk of root perforations, which is more prominent in teeth with narrow, angulated, and curved canals [3, 4]. Also, short clinical crown and decreased occlusogingival height would result in crowns with poor retention and stability [5]. Endocrown restorations obtain their micromechanical retention from the internal (I) pulp chamber walls, and also benefit from chemical retention by bonding to the residual tooth structure by an adhesive cement [2]. Greater preservation of the residual tooth structure, better distribution of the masticatory forces, excellent esthetics, and shorter clinical and laboratory fabrication time all contribute to the increasing popularity of endocrowns as a suitable alternative for reconstruction of endodontically treated molar teeth with extensive coronal destruction [6–8]. Increased cavity depth and extension into the pulp chamber increase the stability, retention, and bonding surface area of endocrown restorations [6]. A specific guideline does not exist for the required minimal extension of cavity depth into the pulp chamber for sufficient retention and resistance [3]. Gaintantzopoulou and El-Damanhoury [9] concluded that a minimum of 2 mm of intracoronal extension would suffice.

Optimal marginal (M) and I adaptation is imperative for the long-term success of restorations and depends on several parameters such as material type, cement type, restoration design, impression technique, and fabrication protocol [10]. Although the reported thresholds are variable, M gaps >120 µm can lead to cement exposure, cement dissolution, and subsequent microleakage, secondary caries development, and gingival inflammation [11, 12]. Moreover, increased polymerization shrinkage and interfacial stress due to greater cement thickness would increase the risk of restoration fracture [13].

The conventional impression technique with elastomeric impression materials has long been used extensively for the fabrication of prosthetic restorations [7]. However, accuracy of the conventional impression technique depends on several parameters such as type of impression material, tray, impression material mixing method, and the transfer process to a laboratory [7]. The digital dental technology and intraoral scanners were introduced aiming to minimize or eliminate the problems related to the dimensional changes of impression materials, dental stone expansion, and human errors associated with the fabrication of master casts [14].

Several studies evaluated the effects of digital and conventional impression techniques on the M and I adaptation of endocrowns and reported conflicting results [7, 8, 10]. Moreover, number of studies on the effect of pulp chamber depth on the accuracy of intraoral scanners is limited [6, 9, 15], and the available ones showed that increased intracoronal and intrapulpal depths decreased M and I adaptation of endocrowns. The aforementioned studies evaluated the effect of cavity depth on adaptation of endocrowns fabricated by the digital impression technique. To the best of the authors' knowledge, no study is available comparing the conventional and digital impression techniques in this respect or showing the superiority of one technique over the other at a specific cavity depth. Thus, the purpose of this study was to compare the I and M adaptation of endocrowns fabricated by the digital and conventional techniques at different cavity depths. The null hypothesis of the study was that the I and M adaptation of endocrowns fabricated by the digital and conventional techniques would not be significantly different at different cavity depths.

## 2. Materials and Methods

In this in vitro study, two mandibular right permanent first molars with no caries or cracks and almost similar buccolingual and mesiodistal dimensions at the cementoenamel junction with a maximum of 10% deviation [16] were used in this study. The teeth had been extracted due to periodontal problems, and were stored in 0.1% thymol solution at room temperature until the experiment. This study was approved by ethics committee of Guilan University of medical sciences [IR.GUMS.REC.1399.562].

**Figure 1:**
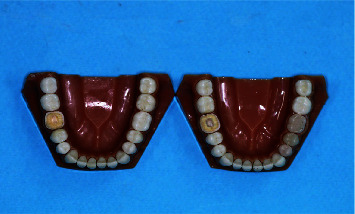
Prepared teeth with 2- and 4-mm cavity depths mounted at the first molar site of dentiform models of mandible.

For specimen preparation, the teeth were initially sectioned perpendicular to their longitudinal axis at 2 mm above their cementoenamel junction and underwent endodontic treatment. They were then mounted in two full-arch dentiform models of mandible at the site of first molar tooth with a similar distance between the prepared tooth margin and the adjacent tooth in the two models. In each tooth, the upper part of the canal over the gutta-percha filling was emptied by a small tungsten carbide bur (Meisinger HM1; Hager and Meisinger, Berlin, Germany) to 1 mm below the orifice and the canal was filled to the level of the pulp chamber floor with flowable composite resin (Denfil Flow; Vericom Laboratories Ltd, Anyang, Korea). The occlusal surface was prepared with a butt-joint margin, and the pulp chamber walls were prepared with 8−10° taper with no undercut by using a round-end tapered diamond bur (856; Drendel + Zweiling Diamant GmbH, Lemgo, Germany). The cavity depth (from the pulp chamber floor to the occlusal surface) was considered to be 2 mm in one tooth and 4 mm in another tooth and checked by a digital caliper. Final finishing and rounding of all I line–angles were performed by a round-end red diamond bur. All preparations were performed by a trained clinician (Figure 1).

Four groups were evaluated in this study, according to the impression technique and cavity depth, comprising a total of 68 specimens with 17 specimens per group: (I) the conventional impression technique with 2 mm cavity depth, (II) the conventional impression technique with 4 mm cavity depth, (III) the digital impression technique with 2 mm cavity depth, and (IV) the digital impression technique with 4 mm cavity depth.

Seventeen conventional impressions were made, and 17 digital scans were obtained from each dentiform model. A prefabricated tray and addition silicone impression material with putty and extra-light consistencies (Panasil, Kettenbach GmbH & Co. KG, Eschenburg, Germany) were used for the conventional impressions, which were made in two steps as instructed by the manufacturer. The obtained impressions were evaluated to ensure absence of irregularities and voids and were then poured with type IV dental stone (GC Fuji Rock EP, GC Europe, Leuven, Belgium). The casts were scanned by a laboratory scanner (CERAMILL MAP 400, Amann Girbach AG, Kolbach, Austria) and the obtained information was saved in standard tessellation language (STL) format. In the digital groups, the scanner was first calibrated with the factory settings, and then digital scans were obtained from the dentiform models with an intraoral scanner (Planscan; Planmeca, Helsinki, Finland). The scan data were then saved in STL format.

**Figure 2:**
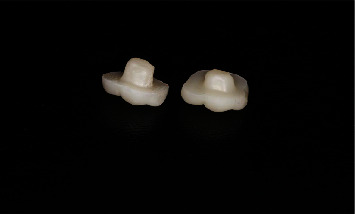
Zirconia endocrowns fabricated by the digital impression technique with 2- and 4-mm cavity depths.

The STL files obtained from conventional and digital impressions were exported to Exocad (Exocad DentalCAD, Exocad GmbH, Darmstadt, Germany) to design endocrown restorations with 50 µm cement space, and equal occlusogingival height [17]. Nonsintered zirconia blocks (Zolid Fx multilayer; Amann Girrbach AG, Kolbach, Austria) were then used for the fabrication of endocrowns in a milling machine (Ceramill Motion 2; Amann Girrbach AG, Kolbach, Austria). They were then sintered using a Ceramill Therm furnace (Ceramill Therm, Amann Girrbach, Koblach, Austria) for 2 h at 1550°C (Figure 2). The fabricated endocrowns were placed on the respective teeth and their seating was checked by using a dental explorer and silicone pressure indicating paste (Fit Checker II, GC, Tokyo, Japan). None of the restorations required any adjustment.

**Figure 3:**
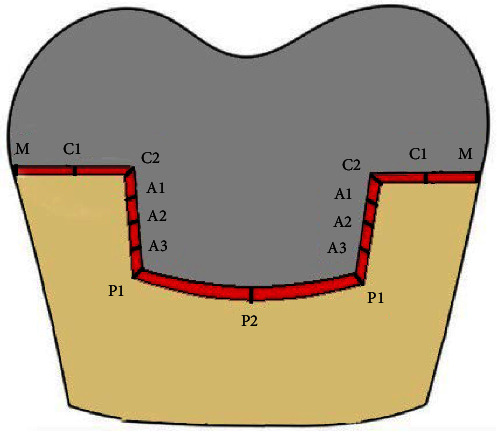
Measurement points for M and I adaptation. Red layer represents cement layer. M1: absolute M discrepancy. C1 and C2: C discrepancies. A1, A2, and A3: A discrepancies. P1 and P2: P discrepancies. A, axial; C, cervical; I, internal; M, marginal; P, pulpal.

The I and M adaptation of endocrowns was evaluated by the silicone replica technique. For this purpose, light-body silicone (Panasil, Kettenbach GmbH & Co. KG, Eschenburg, Germany) was first applied on the I restoration surface and into the pulp chamber of the tooth, and then the restoration was placed on its respective tooth with 50 N force for 5 min using a universal testing machine [18]. After restoration removal, medium-body silicone was injected over the residual silicone material on the tooth surface. The silicone replica was then sectioned mesiodistally and buccolingually with a scalpel, and one slice was obtained from each piece with optimal thickness and parallel walls. Eight points in each slice were selected for the measurement of gap size by using a video measuring machine (C-Class Vision Measurement Machine; Easson optoelectronica technology Co, Suzhou, China) at ×132.8 magnification: one point at the margin (M), two points at the cervical (C) region (C1 at the center and C2 at the cervico-axial angle), three points in the axial (A) wall with equal distances from each other (A1, A2, and A3), and two points at the pulpal (P) floor (P1 at the axio-pulpal angle and P2 at the center of P floor, Figure 3). For the measurement of I and M adaptation, the average values of specific points in each region were used. In the A region, the average was calculated from three points: A1, A2, and A3. In the P region, the average was obtained from two points: P1 and P2. Similarly, in the C region, the average was derived from two points: C1 and C2. The M discrepancy was measured as the distance between the outermost point of the restoration margin and the prepared tooth surface (absolute M discrepancy) [7]. I discrepancy was measured by calculation of the vertical distance between the I crown surface and external preparation surface, except at C2 and P1 which were the bisectors of the cervico-axial and axio-pulpal angles, respectively. These measurements inherently included the cement space since the replicas were made with the endocrowns in their final seated position, simulating the clinical scenario where the cement space is occupied by the luting agent. The magnitude of gap of the endocrowns at the M and I areas (C, A, and P) was separately reported for the buccal (Bu), lingual (Li), mesial (Me), and distal (Di) surfaces in micrometers (µm).

Normal distribution of data was evaluated by the Shapiro–Wilk test, and homogeneity of the variances was analyzed by the Levene test. Comparisons were made by independent sample *t*-test, and analysis of variance (ANOVA) with Tukey test for pairwise comparisons, and also generalized estimating equation with Bonferroni correction for pairwise comparisons. All statistical analyses were performed using statistical package for the social sciences (SPSS) version 26 (SPSS version 26; IBM Corp, NY, USA) at 0.05 level of significance.

## 3. Results

Assessment of the effect of impression technique on the mean gap size (in µm) at each of the M, C, A, P, and I points (Table 1) revealed that the effect of impression technique on the mean gap size was significant at all areas at 2 mm cavity depth (*p*  < 0.001), and at M, C, P, and I at 4 mm cavity depth (*p*  < 0.05), and a smaller mean gap size was recorded in the digital impression technique (*p*  < 0.05). No significant difference was found in the mean gap size at A in 4 mm cavity depth between the digital and the conventional impression techniques (*p*=0.062).  Table 2 presents the results of the comparison of the two techniques separately in the Bu, Li, Me, and Di surfaces.

Assessment of the effect of cavity depth on the mean gap size at M, C, P, A, and I (Table 1) indicated larger gaps in endocrowns with 4 mm cavity depth at P in the conventional technique (*p*=0.008), and at M, A, P, and I (*p*  < 0.001) in the digital technique; however, cavity depth had no significant effect on the gap size at other points (*p*  > 0.05).  Table 2 presents the results regarding the effect of cavity depth on the gap size at different points separately in the Bu, Li, Me, and Di surfaces.

Comparison of the gap size among the Bu, Li, Me, and Di surfaces at each point in the study groups (Table 2) revealed no significant difference among different surfaces in groups II and III (*p*  > 0.05). The difference was significant at points A, C, and I in the group I, and at point A in the group IV (*p*  < 0.05). Pairwise comparisons showed that in both the aforementioned groups, the gap size at point A in the Me surface was significantly greater than that in the Bu surface (*p*  < 0.05). In the group I, the gap size at point C in the Li surface was significantly smaller than that in the Me and Di surfaces (*p*  < 0.05). The difference in gap size was not significant among other surfaces (*p*  > 0.05).

Comparison of the mean gap size at points M, C, P, and A in each group by ANOVA revealed a significant difference (*p*  < 0.001). Pairwise comparisons revealed the largest gap at point P in all groups. In groups I, II, and IV, the smallest gap was noted at points M and C, and the difference in gap size between M and C points was not significant (*p*  > 0.05). In the group III, the smallest gap was noted at point M (*p*  < 0.001), and the difference between C and A points was not significant (*p*=0.993).  Table 3 presents the comparison of gap size at different points in each surface.

## 4. Discussion

The null hypothesis of the study regarding the insignificant effect of the impression technique (digital/conventional) and cavity depth on the M and I gaps was rejected.

The present results regarding the comparison of digital and conventional impression techniques revealed that at both 2- and 4-mm cavity depths, the magnitude of I and M gaps was smaller in the digitally fabricated restorations. Similar results were reported in previous studies comparing the effects of digital and conventional impressions on the I and M adaptation of endocrowns [7]. In the process of conventional fabrication of endocrown restorations, a combination of human errors and other types of errors of impression making and pouring the cast in a laboratory may occur, which can increase the magnitude of M and I gaps of restorations [7]. However, in the fully digital technique, additional laboratory procedures are eliminated. Thus, errors are minimized, and higher adaptation of restoration may be expected [7]. Abduljawad and Rayyan [10] compared the I and M adaptation of lithium disilicate endocrowns fabricated by a fully digital technique and laboratory scanning of the cast obtained from a conventional impression by microcomputed tomography. Unlike the present study, they found no significant difference between the two methods. Also, Falahchai et al. [8] found no significant difference in M gap of zirconia endocrowns fabricated by the digital and conventional impression techniques. It has been stated that the technique of measurement of gap has a direct effect on the results [19]. Thus, differences in the results reported in the literature are justifiable considering no standardization of methods and lack of a standard protocol for this purpose. Furthermore, differences in specimen preparation, materials used for restoration fabrication [16, 20], and fabrication process [21] can all affect the gap size, and limit precise comparison of the present results with previous findings.

In the digitally fabricated restorations, increasing the cavity depth to 4 mm increased the M and I gaps, particularly at the A and P areas. In the conventional impression technique, this increase in gap size only occurred in the P region; in other areas, no significant difference was noted in gap size in comparison with 2 mm cavity depth. To the best of the authors' knowledge, no previous study evaluated the effect of cavity depth on the accuracy of the conventional impression technique. However, the results of previous studies regarding the effect of cavity depth on the accuracy of the digital impression technique were in agreement with the present findings, indicating that increased cavity depth had a negative effect on M and I adaptation of endocrowns fabricated by using an intraoral scanner [6, 15, 22]. One major limitation of using an intraoral scanner is decreased accuracy of recording of each region due to increased depth [6, 9]. By an increase in distance from the scanning surface, the I areas are recorded with lower accuracy. Thus, increased I gap and limitations in precise seating of restoration explain the increase in M gap size. In the present study, increased cavity depth in use of intraoral scanner had no significant effect on adaptation of restorations in the C region. This finding can be explained by no change in the distance between this point and the head of scanner in the tooth with increased cavity depth. Comparison of gap size at different points revealed the largest I gap at the P region. Similar results were reported by previous studies that compared the digital and conventional impression techniques for the fabrication of ceramic endocrowns [7, 10]. Since the complexities and interferences are lower in the A and M areas, compared with the P/occlusal surface, designing and preparation of restoration at these areas are performed with higher accuracy [23]. This finding is further intensified in the conventional impression technique and by dimensional changes of the impression material and the cast [23].

Comparison of the A gap size among the Bu, Li, Me, and Di surfaces revealed the largest gaps in the Me surface in all four groups; however, this difference was only significant in comparison with the Bu surface in the groups I and IV. It has been reported that in digital impression of full-coverage crowns, the Di shadow phenomenon (shadow at the Di of the scanned area) prevents precise recording of this region [24]. Considering the fact that in cavity preparation for an endocrown restoration, the preparation design is the opposite of the preparation performed for a full-coverage crown, lower adaptation of restoration in the Me surface is justified. Nonetheless, since literature is scarce regarding the comparison of gap size at different areas, further studies with a larger sample size are warranted to obtain more accurate results.

In vitro design and not taking into account the confounding factors such as the presence of saliva, blood, and oral contaminants in digital and conventional impression techniques were among the limitations of the present study. Also, the restoration gap was not evaluated after the cementation and aging process, although it would better simulate the clinical setting. Since the space occupied by cement may increase the magnitude of M gap, further studies with a larger sample size are required to take into account the effect of cement on the M and I gaps.

In summary, based on the findings of this in vitro study, it can be concluded that in both groups of restorations with 2- and 4-mm cavity depths, the digital impression technique resulted in smaller M and I gaps than the conventional technique. In the conventional technique, cavity depth had no significant effect on the M and I gaps; however, in the digital technique, increasing the cavity depth to 4 mm increased the M and I gaps. In comparison of Bu, Li, Me, and Di surfaces, the A gap was larger in the Me than the Bu, and the C gap was larger in the Me and Di surfaces than the Li surface only in the groups I and IV. In all groups, the largest gap was noted in the P region and the smallest gap was found in the M and C regions in the groups I, II, and IV and in the M region in the group III.

## Figures and Tables

**Table 1:** Mean and standard deviation of gap size (in µm) and its comparison at the M, C, A, P, and I areas of endocrowns with 2- and 4-mm cavity depths fabricated by the digital and conventional impression techniques.

Region	Cavity depth (mm)	Conventional mean ± SD	Digital mean ± SD	*p* value (F)*⁣*^*∗*^
M	2	101.16 ± 15.92	71.24 ± 15.58	<0.001 (11.08)
	4	103.87 ± 14.15	91.56 ± 14.71	<0.001 (4.97)

*p* value (F)*⁣*^*∗*^	—	0.296 (1.05)	<0.001 (7.82)	—

C	2	103.73 ± 17.14	92.83 ± 13.95	<0.001 (4.07)
	4	108.28 ± 15.39	96.67 ± 14.33	<0.001 (4.55)

*p* value (F)*⁣*^*∗*^	—	0.105 (1.63)	0.116 (1.58)	—

A	2	124.51 ± 16.11	93.56 ± 16.37	<0.001 (11.11)
	4	128.41 ± 14.95	123.71 ± 14.18	0.062 (1.88)

*p* value (F)*⁣*^*∗*^	—	0.146 (1.46)	<0.001 (11.48)	—

P	2	148.68 ± 13.85	130.12 ± 16.27	<0.001 (7.16)
	4	155.70 ± 16.35	147.83 ± 14.36	0.003 (2.98)

*p* value (F)*⁣*^*∗*^	—	0.008 (2.70)	<0.001 (6.73)	—

I	2	125.55 ± 15.59	105.38 ± 15.11	<0.001 (7.66)
	4	130.70 ± 15.82	122.61 ± 15.69	0.003 (2.99)

*p* value (F)*⁣*^*∗*^	—	0.058 (1.91)	<0.001 (6.52)	—

*Note:* Statistically significant at *p*  < 0.05.

Abbreviations: A, axial; C, cervical; I, internal; M, marginal; P, pulpal; SD, standard deviation.

*⁣* ^*∗*^Independent sample test.

**Table 2:** Comparison of gap size (in µm) at the M, C, A, P, and I areas of endocrowns separately in the Bu, Li, Me, and Di surfaces in the study groups (*n* = 17).

Variables	C-2	D-2	*p* value (F)*⁣*^*∗*^	C-4	D-4	*p* value (F)*⁣*^*∗*^
M						
Bu	105.19 ± 16.23^a^	67.93 ± 11.99^a^	<0.001	100.03 ± 13.91^a^	92.14 ± 12.98^a^	0.097
Li	97.22 ± 16.56^a^	72.09 ± 19.63^a^	<0.001	102.03 ± 14.59^a^	89.98 ± 13.89^a^	0.019
Me	99.01 ± 13.18^a^	73.92 ± 14.41^a^	<0.001	108.29 ± 14.39^a^	92.97 ± 16.53^a^	0.07
Di	103.22 ± 17.51^a^	71.01 ± 16.11^a^	<0.001	105.13 ± 13.54^a^	91.16 ± 16.30^a^	0.011
*p* value (F)*⁣*^*∗∗*^	0.409 (2.89)	0.277 (434)	—	0.233 (4.39)	0.935 (0.42)	—
C						
Bu	98.63 ± 12.98^ab^	87.24 ± 12.26^a^	0.013	105.74 ± 12.94^a^	95.35 ± 17.44^a^	0.057
Li	92.94 ± 16.76^a^	94.94 ± 11.76^a^	0.690	110.45 ± 12.27^a^	92.76 ± 14.63^a^	0.001
Me	112.58 ± 16.49^b^	96.20 ± 14.58^a^	0.004	108.97 ± 16.55^a^	99.18 ± 12.80^a^	0.063
Di	110.75 ± 14.93^b^	92.92 ± 16.25^a^	0.002	107.96 ± 19.68^a^	99.38 ± 12.06^a^	0.137
*p* value (F)*⁣*^*∗∗*^	0.002 (15.39)	0.074 (6.93)	—	0.740 (1.25)	0.355 (3.25)	—
A						
Bu	118.09 ± 14.54^a^	92.85 ± 20.21^a^	<0.001	126.73 ± 14.62^a^	119.74 ± 12.17^a^	0.140
Li	120.11 ± 14.81^ab^	88.37 ± 14.03^a^	<0.001	125.65 ± 15.16^a^	121.84 ± 14.38^a^	0.458
Me	133.46 ± 17.52^b^	97.58 ± 13.20^a^	<0.001	131.17 ± 16.29^a^	129.36 ± 14.66^a^	0.736
Di	126.38 ± 14.01^ab^	95.46 ± 17.11^a^	<0.001	130.10 ± 14.32^a^	123.89 ± 10.64^a^	0.222
*p* value (F)*⁣*^*∗∗*^	0.017 (10.14)	0.279 (3.84)	—	0.761 (1.16)	0.012 (10.94)	—
P						
Bu	149.52 ± 13.57^a^	131.82 ± 14.74^a^	0.001	156.43 ± 15.80^a^	146.24 ± 15.25^a^	0.065
Li	145.23 ± 13.58^a^	126.78 ± 17.20^a^	0.002	153.94 ± 19.57^a^	144.89 ± 15.55^a^	0.121
Me	148.43 ± 12.70^a^	133.34 ± 19.80^a^	0.013	155.37 ± 19.57^a^	151.78 ± 15.73^a^	0.559
Di	151.55 ± 15.85^a^	128.54 ± 13.22^a^	<0.001	157.07 ± 13.21^a^	148.42 ± 10.64^a^	0.044
*p* value (F)*⁣*^*∗∗*^	0.538 (1.95)	0.678 (1.52)	—	0.905 (0.56)	0.710 (1.38)	—
I						
Bu	122.07 ± 15.30^a^	103.81 ± 14.18^a^	0.001	129.46 ± 15.41^a^	120.61 ± 17.63^a^	0.129
Li	119.44 ± 17.15^a^	103.49 ± 14.69^a^	0.007	129.79 ± 13.75^a^	119.80 ± 13.53^a^	0.040
Me	131.38 ± 16.85^a^	109.07 ± 17.49^a^	0.001	131.90 ± 16.29^a^	126.35 ± 14.10^a^	0.296
Di	129.31 ± 10.06^a^	105.16 ± 14.56^a^	<0.001	131.65 ± 18.74^a^	123.69 ± 17.61^a^	0.211
*p* value (F)*⁣*^*∗∗*^	0.017 (10.15)	0.789 (1.01)	—	0.951 (0.35)	0.490 (2.43)	—

*Note:* Statistically significant at *p*  < 0.05. Values with different superscript lowercase letters in the same column are significantly different (*p*  < 0.05).

Abbreviations: A, axial; Bu, buccal; C, cervical; C-2, conventional technique-2-mm cavity depth; C-4, conventional technique-4-mm cavity depth; D-2, digital technique-2-mm cavity depth; D-4, digital technique-4-mm cavity depth; Di, distal; I, internal; Li, lingual; M, marginal; Me, mesial; P, pulpal; SD, standard deviation.

*⁣* ^*∗*^Independent sample *t*-test.

*⁣* ^*∗∗*^Generalized estimating equation and Bonferroni correction post hoc test.

**Table 3:** Comparison of gap size (µm) at the M, C, A, P, and I areas separately in the Bu, Li, Me, and Di surfaces in the study groups (*n* = 17).

Variables	M	C	A	P	*p* value (F)*⁣*^*∗*^
C-2					
Bu	AB	A	B	C	<0.001 (41.98)
Li	A	A	B	C	<0.001 (41.09)
Me	A	A	B	C	<0.001 (35.70)
Di	A	A	B	C	<0.001 (31.72)
D-2					
Bu	A	B	B	C	<0.001 (53.07)
Li	A	B	B	C	<0.001 (35.18)
Me	A	B	B	C	<0.001 (41.63)
Di	A	B	B	C	<0.001 (38.62)
C-4					
Bu	A	A	B	C	<0.001 (53.77)
Li	A	A	B	C	<0.001 (39.34)
Me	A	A	B	C	<0.001 (29.97)
Di	A	A	B	C	<0.001 (41.49)
D-4					
Bu	A	A	B	C	<0.001 (50.39)
Li	A	A	B	C	<0.001 (53.85)
Me	A	A	B	C	<0.001 (56.71)
Di	A	A	B	C	<0.001 (61.27)

*Note:* Values with different uppercase letters in the same row are significantly different (*p*  < 0.05).

Abbreviations: A, axial; Bu, buccal; C, cervical; C-2, conventional technique-2-mm cavity depth; C-4, conventional technique-4-mm cavity depth; D-2, digital technique-2-mm cavity depth; D-4, digital technique-4-mm cavity depth; Di, distal; I, internal; Li, lingual; M, marginal; Me, mesial; P, pulpal.

*⁣* ^*∗*^Generalized estimating equation with Bonferroni correction post hoc test.

## Data Availability

The datasets used and/or analyzed during the current study are available from the corresponding author on reasonable request. Also, the datasets supporting the conclusions of this article are included within the article.
